# Capillary Forces between Concave Gripper and Spherical Particle for Micro-Objects Gripping

**DOI:** 10.3390/mi12030285

**Published:** 2021-03-08

**Authors:** Zenghua Fan, Zixiao Liu, Congcong Huang, Wei Zhang, Zhe Lv, Lefeng Wang

**Affiliations:** 1 School of Mechanical Engineering, Shandong University of Technology, Zibo 255049, China; sdut2015lzx@163.com (Z.L.); sdut2020hcc@163.com (C.H.); zw062003@163.com (W.Z.); lzjslyz@126.com (Z.L.); 2 State Key Laboratory of Robotics and System, Harbin Institute of Technology, Harbin 150080, China; lefengwang@hit.edu.cn

**Keywords:** capillary bridge, capillary force, concave gripper, micromanipulation

## Abstract

The capillary action between two solid surfaces has drawn significant attention in micro-objects manipulation. The axisymmetric capillary bridges and capillary forces between a spherical concave gripper and a spherical particle are investigated in the present study. A numerical procedure based on a shooting method, which consists of double iterative loops, was employed to obtain the capillary bridge profile and bring the capillary force subject to a constant volume condition. Capillary bridge rupture was characterized using the parameters of the neck radius, pressure difference, half-filling angle, and capillary force. The effects of various parameters, such as the contact angle of the spherical concave gripper, the radius ratio, and the liquid bridge volume on the dimensionless capillary force, are discussed. The results show that the radius ratio has a significant influence on the dimensionless capillary force for the dimensionless liquid bridge volumes of 0.01, 0.05, and 0.1 when the radius ratio value is smaller than 10. The effectiveness of the theorical approach was verified using simulation model and experiments.

## 1. Introduction

Capillary actions between micro- and nanoparticles have aroused much attention because of the ubiquitous presence of liquid bridge systems with a variety of applications in the pharmaceutical, chemical, cosmetic, and agricultural industries. Compared to the force of gravity, capillary forces play a critical role in micro- and nanometer scales because of the scaling effects, especially in particle wetting, lubrication, and self-alignment [1,2].

As a dominant force at the micro- and nanoscale, capillary action can be utilized as a gripping mechanism. A typical example of capillary applications can be the so-called micromanipulation technique, which has been widely used in micro-objects gripping. The formed liquid bridge is employed as a flexible tool for micro-objects manipulation, which contributes to avoiding stress concentration when compared to the mechanical microgripper. Vasudev et al. [3] designed an electrowetting-based capillary microgripper, in which the manipulated objects were controlled by changing the contact angle between the liquid bridge and the gripper surface under the action of electrowetting. Experiments demonstrated that various micro glass beads ranging from 77 to 136 µN were picked by varying the applied voltage. As a further application, an ionic liquid (BmimPF6) droplet was used as the operating liquid for the electrowetting-based capillary microgripper was demonstrated in environment with a maximum temperature of 110 °C [4]. Lambert et al. [5] developed a capillary microgripper, using the effects of the surface tension to pick and place a watch bearing with a submillimetric size, which is well adapted for the handling of 0.3 mm and 0.5 mm diameter balls. Fantoni el al. [6] proposed a novel capillary manipulating strategy for grasping and releasing mini- and microparts by changing the transition of liquid between hydrophobic and hydrophilic surfaces. Microspheres varying from 0.5 mm to 2.85 mm, and flat components such as metal mini-screws, were manipulated to verify the proposed method. To achieve the orientation adjustment of the manipulated objects in capillary action, Zhang et al. [7] designed a manipulator with multi-needle. The shape of the liquid droplet was changed by adjusting the relative position of each needle, resulting in the micro-components tilting and their rotation. Fan et al. [8,9] proposed a capillary gripping method based on dropwise condensation to guarantee real-time droplet formation. Microspheres with 40–200 μm diameter were grasped reliably using a single-probe capillary microgripper with hydrophobicity.

For particles and devices in microscales, the capillary action was also widely employed in micro-objects self-assembly. Li et al. [10] analyzed the self-folding of thin plates into deterministic 3D shapes based on liquid bridge interaction. Similarly, the self-alignment of SU-8 microchips on the patterned substrate with an oleophilic/oleophobic surface was investigated using adhesive droplets, which help to achieve a constant volume of low evaporation rate in room temperature [11]. Compared to square chip alignment, Berthier et al. [12] established the capillary-driven model for the self-alignment of polygonal chips. The shift-restoring forces for various configurations including regular convex and nonconvex polygons were investigated using the Surface Evolver (SE) software. In our previous works, the self-alignment release of microspheres with diameters of 100 µm and 200 µm was implemented on the basis of water condensation [13]. The adhered microspheres were released on the target position because of the capillary action of the formed liquid bridge.

So, it is important to develop general expressions of the liquid bridge for predicting the capillary force. Wang el al. [14] modelled the axisymmetric liquid bridge between two continuously fully wet disks. Capillary forces with various liquid bridge heights were investigated by the energy minimization method for a constant liquid volume. Profiles of the capillary bridges between a rough surface and a parallel surface were modelled [15], and the predicted results were verified experimentally. Ataei et al. [16] performed systematic investigations on a capillary bridge between nonparallel solid surfaces by combining experimental and numerical approaches. The critical dihedral angle ensuring the stability of the formed capillary bridge was significantly determined by the advancing contact angle and contact angle hysteresis. Wang et al. [17] employed the iterative method to estimate the capillary forces between two axisymmetric power–law particles at a fixed liquid volume.

To predict the profile of a liquid bridge between two spherical particles, Wang et al. [18] proposed an asymptotic solution method based on a rapid convergent predictor–corrector algorithm, in which the minimal surface ensures the minimum total surface energy. Nguyen et al. [19] investigated an original approach for measuring surface tension from two equal-sized spherical particles. The meniscus profile of the capillary bridge was recorded using a high-resolution camera to calculate the capillary force. A minimal energy method was employed to explore the rupture of the liquid bridge between two unequal particles by means of the software Surface Evolver [20]. The effects of the particle radius ratio, the contact angle, and the liquid bridge volume on the liquid transfer ratio were examined in detail. Furthermore, Tourtit et al. [21] focused on the experimental study of the rupture of an axially symmetric liquid bridge between a cone and a plane. The capillary force applied on a tilted cylinder was measured using a customized atomic force microscope (AFM) probe to investigate the relationship between the capillary force and the dipping angle [22].

To improve the gripping ability, a concave-shaped probe was developed, which shows a much larger capillary force than a flat one [23]. However, few works were performed on the investigations of capillary bridges between a concave surface and a spherical particle. In the present study, capillary bridges between a spherical concave gripper and a spherical particle are investigated in detail. A theoretical point based on the Young–Laplace equation was adopted for the modelling of the capillary bridge and capillary force, subject to the constant volume condition. Parameter changes during the liquid bridge rupture were demonstrated. The effects of the contact angle, the radius ratio, and the liquid volume on the capillary force were analyzed and are discussed in this paper. Finally, simulated and experimental studies were conducted to investigate the effectiveness of the proposed model.

## 2. Modeling the Capillary Bridge and Capillary Force

### 2.1. Geometry of the Capillary Bridge

**Figure 1:**
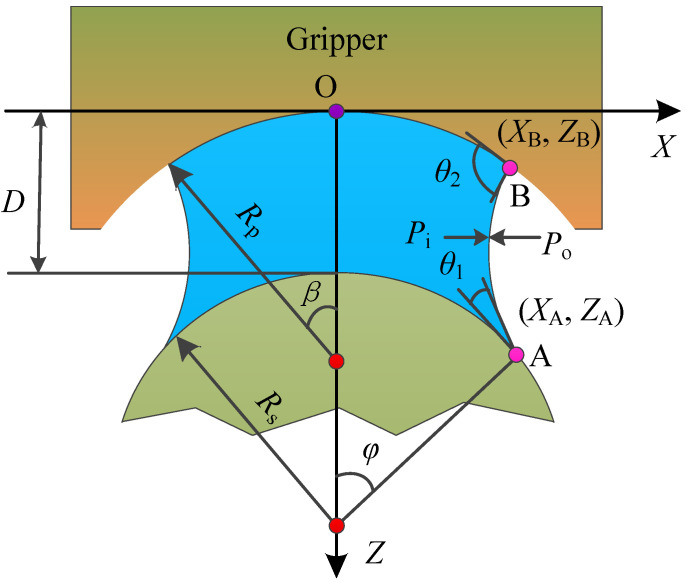
Geometry of the capillary bridge between a concave gripper and a spherical particle.

Figure 1 shows the geometry of the axisymmetric capillary bridge between a concave gripper and a spherical particle. A meniscus is created because of the pressure difference between the outside pressure ($P_{o}$) and inside pressure ($P_{i}$). The axial coordinate along the axis of symmetry is denoted by Z. Accordingly, the meniscus profile of the formed capillary bridge is denoted by X(Z), which represents the local radius of the capillary bridge. $\theta_{1}$ and $\theta_{2}$ are the contact angle of the formed liquid bridge on the spherical particle and the spherical concave, respectively. Coordinates of the contact points caused by the liquid wetting on the spherical particle and the spherical concave surface are A ($X_{A}$, $Z_{A}$) and B ($X_{B}$, $Z_{B}$), respectively. The dimensionless coordinate system is used by scaling with the sphere radius ($R_{s}$) for the description of the liquid bridge. So, the value of the sphere radius is 1 in the dimensionless coordinate system. The dimensionless values of the coordinates ($x_{A}$, $z_{A}$), ($x_{B}$, $z_{B}$) can be derived by the following equations.

(1) $$z_{A}=d+1-\cos\varphi \;\;\;\;\;x_{A}=\sin\varphi$$

(2) $$z_{B}=r_{p}-r_{p}\cos b\;\;\;\;\;x_{B}=r_{p}\sin b$$

where, $d=D/R_{s}$ is the dimensionless separation distance, $\;r_{p}=R_{p}/R_{s}$ is the dimensionless radius of the spherical concave, $R_{p}$ is the radius of the spherical concave surface, φ is the half-filling angle of the particle, and β is the half-filling angle of the concave gripper.

The dimensionless liquid volume (V) with a fixed distance between the particle and the concave gripper is derived using the geometrical relationship as follows:

(3) $$V=\int_{z_{B}}^{z_{A}}\pi x^{2}dz-\pi /3{\left(1-\cos\varphi \right)}^{2}(2+\cos\varphi )+\pi /3r_{p}{}^{3}{\left(1-\cos\beta \right)}^{2}(2+\cos\beta ).$$

Calculating the capillary bridge is critical for solving the meniscus profile. The iterative method was employed for the solution, as described in Section 3.

### 2.2. Capillary Force Based on Young–Laplace Equation

The capillary forces $F_{cp}$, created by the capillary bridge, serve a critical role for bonding particles in micromanipulation tasks. Generally, the capillary force acting on a sphere consists of two parts. One is the pressure force $F_{p}$, arising from the pressure difference ($\Delta P=P_{o}-P_{i}$). The other is the vertical component of the surface tension force $F_{s}$, acting on the particle. Therefore, the capillary force acting on a sphere can be expressed as

(4) $$F_{cp}=F_{p}+F_{s}=\pi {\left(R_{s}\sin\varphi \right)}^{2}\Delta P+2\pi \gamma R_{s}\sin\varphi \sin(\varphi +\theta_{1})$$

where γ is the surface tension of the liquid.

The pressure difference of a symmetric capillary bridge can be derived by the Young–Laplace equation. Various works have shown experimentally that the Young–Laplace equation describes the properties of capillary bridges accurately without the gravity effect [24]. To simplify the calculation of the capillary bridge profile, a dimensionless Young–Laplace equation is written as follows:

(5) $$\frac{x^{\prime \prime }}{{\left(1+x^{\prime^{2}}\right)}^{3/2}}-\frac{1}{x{\left(1+x^{\prime^{2}}\right)}^{1/2}}=\frac{\Delta PR_{s}}{\gamma }=\Delta p$$

where $x^{\prime }=dx/dz$, $x^{\prime \prime }=d^{2}x/dz^{2}$, and Δp is the dimensionless pressure difference. By substituting Equation (5) into Equation (4), the dimensionless capillary force, $f_{cp}$, can be calculated based on the actual capillary force, $F_{cp}$, by scaling with $R_{s}\gamma$, as shown in Equation (6).

(6) $$f_{cp}=F_{cp}/(R_{s}\gamma )=\pi {\left(\sin\varphi \right)}^{2}\Delta p+2\pi \sin\varphi \sin(\varphi +\theta_{1})$$

The capillary force is of essence in solving a second-order differential equation. Two boundary conditions are obtained at the three-phase contact lines of the concave gripper, and the particles are as described in Equation (7) and Equation (8), respectively.

(7) $$x^{\prime }|{}_{z=z_{B}}=1/\tan(\theta_{2}-\beta )$$

(8) $$x^{\prime }|{}_{z=z_{A}}=-1/\tan(\varphi +\theta_{1})$$

The above boundary conditions must satisfy $x^{\prime }=0$, while $\theta_{2}-\beta =\pi /2$ or $\varphi +\theta_{1}=\pi /2$.

## 3. Numerical Solution of Capillary Forces

**Figure 2:**
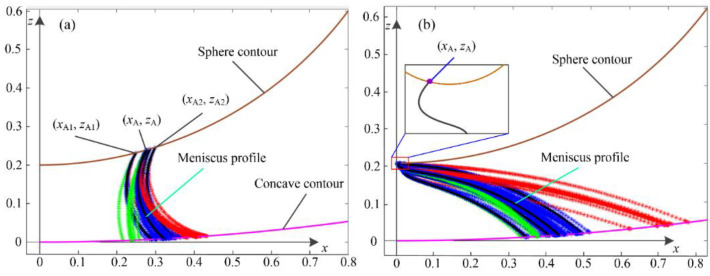
Double iterative processes of meniscus profiles: (**a**) stable solution; (**b**) unstable solution.

To determine the profile of the formed capillary bridge, geometric approximation methods are generally employed to simplify the calculation processes in the absence of gravity, in which the shape of the axisymmetric liquid bridge is fitted as a part of a circular arc or an ellipse [25]. Geometric approximation brings a small error (<10%) with respect to the exact solution of the Young–Laplace equation because of the geometric errors [26,27]. Numerical solutions of the nonlinear Young–Laplace equation are effectively exact, as reported in previous works [25,28]. In present study, a numerical procedure was developed based on a shooting method to obtain the capillary bridge profile and solve the capillary force between a spherical concave gripper and a spherical particle. The parameters of separation distance, liquid volume, and contact angles were assumed to be known as fixed values for a static capillary bridge. The effectiveness of the shooting method was verified by solutions of a capillary bridge between a spherical gripper and a plane, and two axisymmetric power–law particles [17,29]. Two local radii, $x_{A1}$ and $x_{A2}$, of the capillary bridge on the sphere contour ($R_{s}=1$ for dimensionless description) were first given, as shown in Figure 2, and the starting points can be obtained by calculating $z_{A1}$ and $z_{A2}$, according to the geometry equation of the sphere and the separation distance as follows:

(9) $$\{{}_{z_{A2}=d+1-\sqrt{1-{\left(x_{A2}\right)}^{2}}}^{z_{A1}=d+1-\sqrt{1-{\left(x_{A1}\right)}^{2}}}.$$

Two pressure differences ($\Delta p_{1}$, $\Delta p_{2}$) were given for further solution. The contact angle, $\theta_{2}$, at the three-phase contact line on the concave contour was obtained by solving Equation (5) based on the given parameters of $x_{A1}$ and $\theta_{1}$. The two candidate values of $\theta_{2}$ were obtained accordingly. If the target values of $\theta_{2}$ were included the range of the two candidate values, a dichotomous search method was adopted to adjust the profile until the calculated $\theta_{2}$ was the given one, leading to a candidate volume of $V_{1}$. Similarly, the other capillary bridge volume $V_{2}$ was obtained while using the given value of $x_{A2}$ to meet the given contact angle of $\theta_{2}$. If the given volume V was included between $V_{1}$ and $V_{2}$, the formed capillary bridge profile was further adjusted to meet the given volume value. The final local radius, $x_{A}$, of the capillary bridge on the particle was obtained when the profile of the capillary bridge was determined.

Figure 2 shows samples of the double iterative processes in detail between a spherical concave gripper and a sphere. Two solutions of the capillary bridge profile may exist by solving the nonlinear Young–Laplace equation based on the proposed numerical approach, as shown in Figure 2a,b, respectively. The obtained profiles exhibit a stable state and an unstable state in physical relevance. Figure 2a shows a stable meniscus profile with a determined contact point ($x_{A}$, $z_{A}$). As shown in Figure 2b, the curve of the formed meniscus profile changes drastically in the region near the contact point ($x_{A}$, $z_{A}$), where the formed profile transitions from concave shape to convex shape. The related capillary bridge profile does not exist based on the principle of minimum energy, which represents an unstable solution.

## 4. Results and Discussion

In this section, the capillary bridge rupture between a spherical concave surface and a spherical particle is discussed first. The effects of various parameters, including the contact angle of the spherical concave, the radius ratio, and the capillary bridge volume, on the capillary force are investigated in detail.

### 4.1. Capillary Bridge Rupture

**Figure 3:**
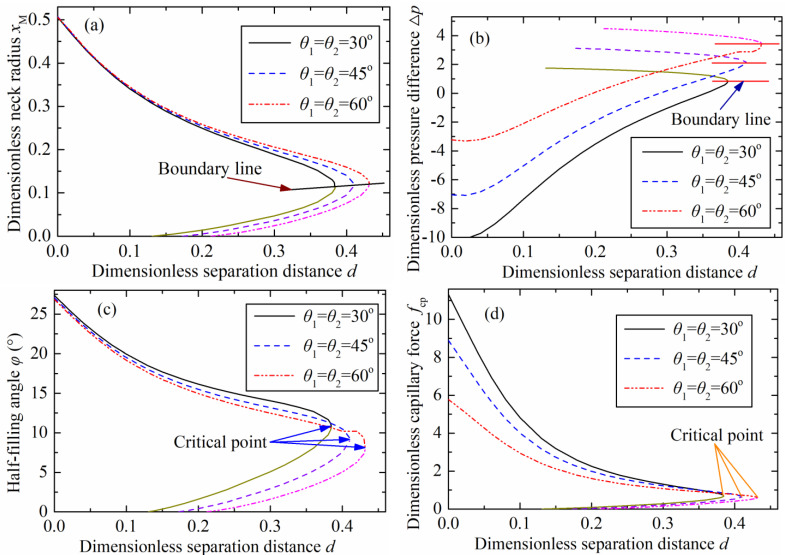
Various parameters changing with dimensionless separation distance variation at three contact angles: (**a**) dimensionless neck radius, (**b**) dimensionless pressure difference, (**c**) half-filling angle, and (**d**) dimensionless capillary force.

Figure 3 plots the changes of various parameters including the neck radius, pressure difference, half-filling angle on the particle, and capillary force with the increasing of separation distance, or the liquid bridge height. Two branches were formed because there were two solutions based on the numerical approaches for obtaining the capillary bridge profile. A stable branch was formed by stable solutions, and the unstable branch plotted as a short line was formed by the unstable solutions, as shown in Figure 3. The two solutions converged to a single solution at a critical separation distance, representing the maximum value of the dimensionless liquid bridge height. No solutions existed if the separation distance exceeded the critical value corresponding to the rupture point.

The critical separation distances were 0.38, 0.41, and 0.43 with contact angles ($\theta_{1}=\theta_{2}$) of 30°, 45°, and 60°, respectively, as shown in Figure 3a. The critical dimensionless separation distances were identical with the results in Figure 3b–d at the same contact angles. This indicates that these rupture criteria are applicable for an axisymmetric capillary bridge between a spherical concave surface and a sphere.

The plotted lines based on the stable solutions were above the boundary line, as shown in Figure 3a. The neck radius decreased as the separation distance increased. As shown in Figure 3b, the stable branches were below the boundary line, in which the pressure difference increased with the increasing of the separation distance. The minimum half-filling angle on the particle was obtained at the critical point of the stable solutions, as shown in Figure 3c. The half-filling angle was equivalent to the dimensionless immersing radius (sinφ). The minimum half-filling angle increased with the contact angle increasing. The variation of the dimensionless capillary force with the separation distance changing at the three representative contact angles (30°, 45°, and 60°) is illustrated in Figure 3d. The maximum capillary force appeared at zero separation distance. There was not capillary force if the separation distance exceeded the critical point. This indicates that the capillary bridge disappears then, also called the capillary bridge rupture.

### 4.2. Capillary Forces

Capillary force plays a critical role while the liquid bridge is employed as a flexible tool, during which the micro-object is picked and transferred to a target point. In this section, the effects of various parameters on the capillary force between a spherical concave gripper and a spherical particle are discussed in detail. The capillary force mainly depends on the contact angle, the radius ratio ($R_{p}/R_{s}$), and the liquid bridge volume. Only stable solutions are discussed in this section.

**Figure 4:**
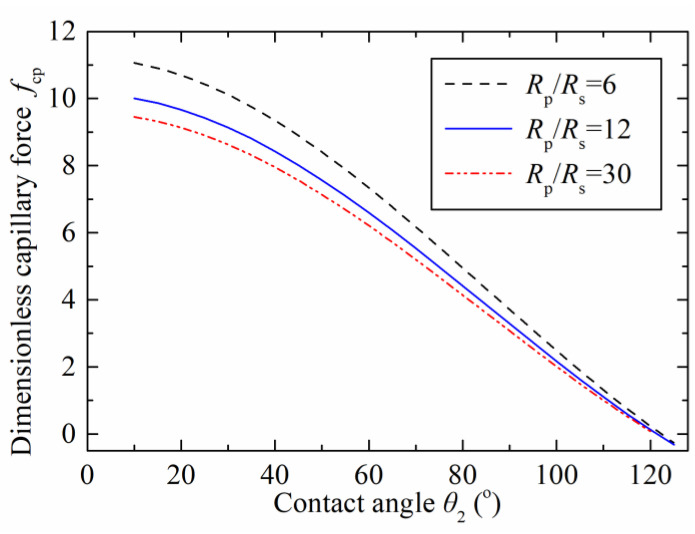
Effects of contact angle on the dimensionless capillary force.

Figure 4 shows the relationship between the capillary force and the contact angle on the concave surface with different radius ratios ($R_{p}/R_{s}$). The adopted contact angle of $\theta_{1}$ was 45° and referred to the liquid-solid contact angle on the particle, the dimensionless capillary bridge volume was 0.05, and the separation distance was zero. The dimensionless capillary force decreased as the contact angle increased. The maximum dimensionless capillary forces were 11, 10, and 9.5 with respect to the radius ratios of 6, 12, and 30 at the contact angle ($\theta_{2}$) of 10°, respectively. The capillary force reduced to zero at the contact angle ($\theta_{2}$) of 120°. This characteristic is caused by the hydrophobicity on the spherical concave surface when the contact angle increases. In this case, the solid surface had difficulty capturing any of the liquid, finally resulting in a repulsive force between the concave gripper and the particle. The results show that the capillary force can be varied by the radius ratio.

**Figure 5:**
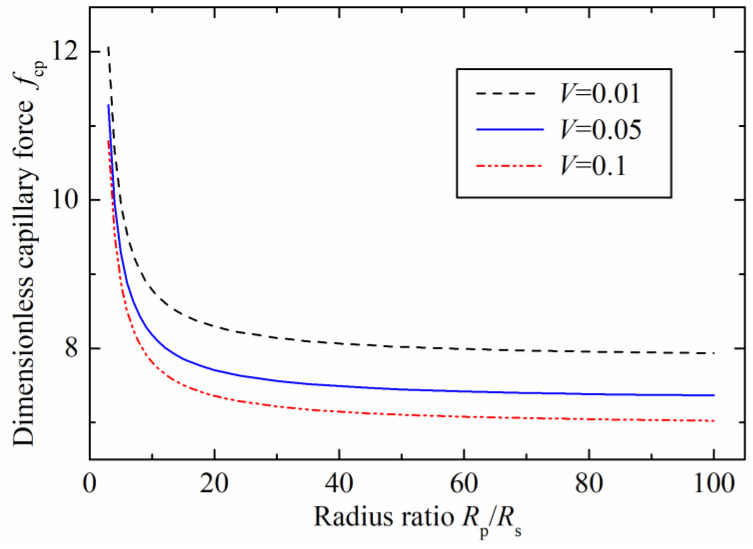
Effects of the radius ratio on the dimensionless capillary force.

The relationships between the dimensionless capillary force and the radius ratio with different dimensionless liquid bridge volume are plotted in Figure 5. The contact angles were 45° on the two surfaces ($\theta_{1}=\theta_{2}=45{}^{\circ}$), and the separation distance was zero. The results demonstrate that the capillary force decreases as the radius ratio increases. This is because the liquid bridge radius on the concave surface gradually increases with the ratio radius, which leads to the increasing of the half-filling angle (β) and contact angle ($\theta_{2}$) accordingly. Therefore, the capillary force experiences a decline as the contact angle on the concave surface increases. The capillary forces were sensitive to the radius ratio in the initial stage ($R_{p}/R_{s}<10$). For the liquid bridge volume of 0.1, the dimensionless capillary force reduced to 7.8 from the initial 10.8 when the radius ratio increased to 10 from the initial ratio of 3. The capillary force kept decreasing when the radius ratio was larger than 20, but the curve of the stable capillary force became flatter, and the capillary force tended to be a constant. Additionally, a stable capillary force of 7 was obtained when the radius ratio reached 100. Therefore, the results also show that the capillary force depends not only on the radius ratio, but also on the liquid bridge volume.

The influence of the dimensionless liquid volume on the dimensionless capillary force with the three radius ratios at zero separation distance was further investigated when 

**Figure 6:**
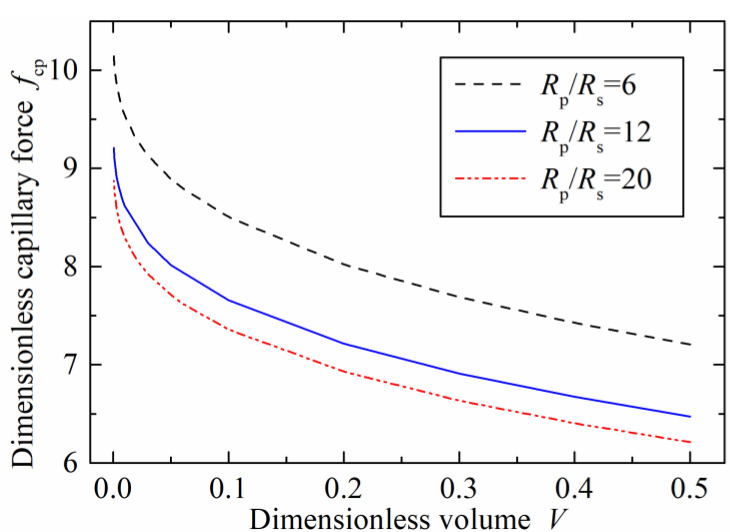
Effects of the dimensionless volume on the dimensionless capillary force.

$\theta_{1}=\theta_{2}=45{}^{\circ}$, as demonstrated in Figure 6. The capillary force decreased sharply with the increasing liquid bridge volume in the initial stage smaller than 0.1. This changing trend was consistent with the capillary interactions between the two rigid spheres, two power–law particles, and plane-sphere [30]. However, the model between the concave surface and sphere showed a big capillary force compared with the models of two sphere interaction or plane-sphere liquid bridge. This implies that a concave microgripper contributes to enhance the gripping ability in micromanipulation tasks.

**Figure 7:**
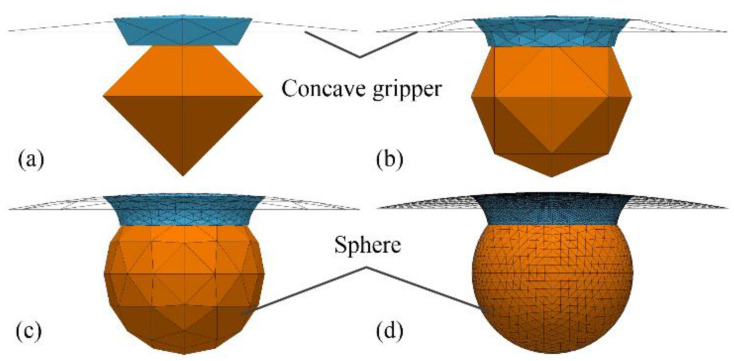
Evolution processes of the capillary bridge: (**a**) initial definition, (**b**,**c**) evolution processes, and (**d**) after evolution.

An alternative approach was employed to estimate the capillary force using the minimum energy method by means of the software Surface Evolver [31], which also served as criteria to verify the effectiveness of the numerical solution method based on the nonlinear Young–Laplace equation. Figure 7 shows the evolution processes of the established capillary bridge model between a spherical concave surface and a spherical particle from an initial arbitrary geometry. The surfaces of the liquid bridge, the concave gripper, and the sphere were defined as collection of triangles connected in an arbitrary topology, as shown in Figure 7a. The total energy was calculated as a function of the surface tension and the coordinates of the vertices of the triangles. The motion of each vertex corresponding to minimum surface energy was managed with a gradient descent optimization algorithm based on the constraints, such as the contact angles and the liquid volume. Capillary bridge evolution was achieved with the motion of all the vertex calculations using an iterative method, as depicted in Figure 7b,c. The surface evolution was finished when the absolute difference of the total energy between the two iterations was smaller than 10^−6^, as illustrated Figure 7d.

**Figure 8:**
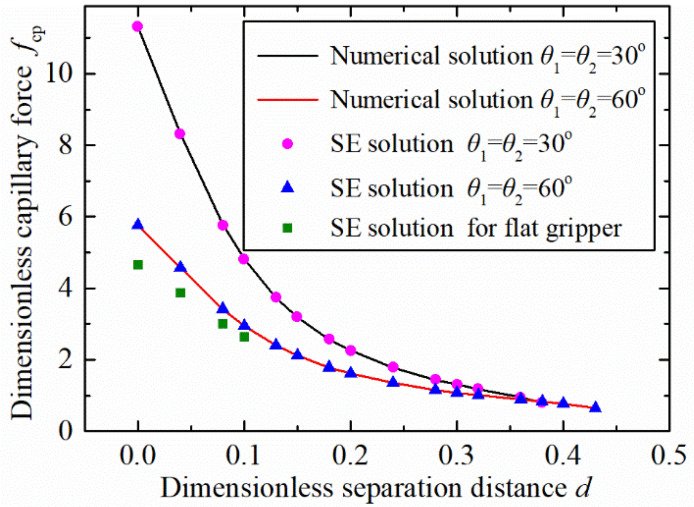
Comparation results between numerical and Surface Evolver (SE) solutions.

The capillary force variation, with the separation distance based on the two adopted solution approaches of the numerical solution and the software Surface Evolver, is shown in Figure 8. The results show that the data obtained by the two methods exhibited good agreement. This indicates that the established model and the solution method are effective to predict the capillary bridge and the capillary between a spherical concave gripper and a spherical particle. Furthermore, the concave shaped gripper showed a larger capillary force than a flat one when $\theta_{1}=\theta_{2}=60{}^{\circ}$.

### 4.3. Experimantal Measurements

**Figure 9:**
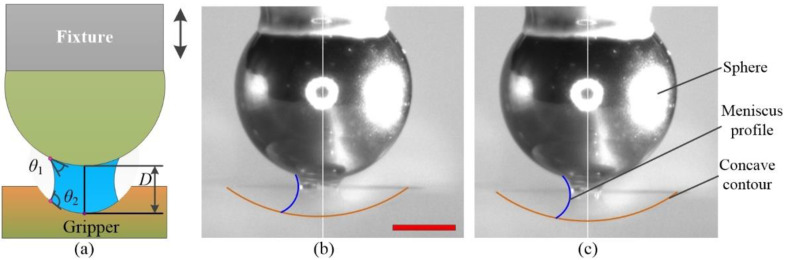
Processes of capillary force measurements: (**a**) geometrical representation of a stable liquid bridge, (**b**) separation distance of 0.36 mm, and (**c**) separation distance of 0.4 mm. The scale bar is 0.5 mm.

An experimental setup was developed to measure the capillary forces of the formed liquid bridge. The spherical concave gripper was made of an acrylic plate and was placed on an electronic microbalance (Sartorius QUINTIX35-1CN) with a 0.01 mg resolution. A stainless-steel ball with a diameter of 1.5 mm, serving as spherical particle, was fixed on a fixture by glue. The vertical motion of the microsphere was provided by a three-axis precision stage, as shown in Figure 9a. A high-resolution camera (Myutron HMZ0745) was positioned on the side view, which obtained the image of the liquid bridge in real time. The back lighting was guaranteed by a LED. The geometrical parameters were manipulated in pixels and then were converted to millimeters. Glycerin was adopted as the liquid for the experimental measurements with a surface tension of 0.063 N/m at 20 °C. A liquid bridge was formed on the basis of the established setup, as shown in Figure 9a,b. Liquid bridges were observed because of the transparency of the acrylic.

**Figure 10:**
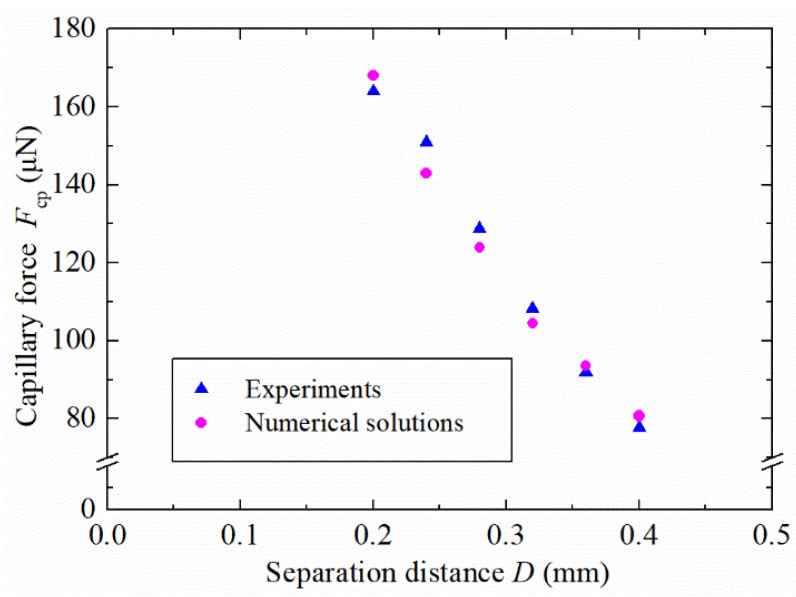
Comparation results between the numerical solutions and experiments.

Experiments were also carried out on glycerin bridges at six separation distances, as shown in Figure 10. For each separation distance, a unique image of the capillary bridge was obtained and processed. The provided data of images were used for numerical calculations. The initial volume of the liquid bridge was 103 nL. Each experiment was repeated three times, and an average of the three trials was plotted in Figure 10. The results exhibit good agreement with the numerical solutions, which verifies the effectiveness of the numerical solution method and the simulation model. The reported results provide a promising solution for capillary forces between a spherical concave gripper and a spherical particle. This can lead to potential applications in micromanipulation and micro-assembly.

## 5. Conclusions

In present work, the evolution of a capillary bridge between a spherical concave gripper and a sphere was investigated. A capillary force model was developed and validated considering the capillary bridge evolution to calculate the capillary force based on the Young–Laplace equation. The capillary bridge profile and the capillary force were obtained by using a numerical procedure based on a shooting method, which consists of double iterative loops. The changes of various parameters, including the neck radius, pressure difference, half-filling angle, and capillary force with the increasing of the separation distance, were performed in detail. The stable and unstable solutions converged to a single solution at a critical separation distance, representing the maximum value of the dimensionless liquid bridge height. The capillary forces, which depend on the contact angle ($\theta_{2}$), the radius ratio, and the liquid bridge volume, were discussed comprehensively using stable solutions. An increase in the contact angle on the spherical concave gripper, the radius ratio, and the liquid bridge volume led to the decrease of the dimensionless capillary force. The effectiveness of the solution method based on the nonlinear Young–Laplace equation was verified using the minimum energy method and various experimental approaches. The reported results provide a promising solution for capillary bridges and capillary forces between a spherical concave gripper and a spherical particle. This can lead to important guidance in micromanipulation and micro-assembly.

## Data Availability

The data presented in this study are available on request from the corresponding author.
